# From Antiretroviral to Antibacterial: Deep-Learning-Accelerated Repurposing and In Vitro Validation of Efavirenz Against Gram-Positive Bacteria

**DOI:** 10.3390/molecules30142925

**Published:** 2025-07-10

**Authors:** Ezzeldin Saleh, Omar A. Soliman, Nancy Attia, Nouran Rafaat, Daniel Baecker, Mohamed Teleb, Abeer Ghazal, Ahmed Noby Amer

**Affiliations:** 1 Microbiology Department, Medical Research Institute, Alexandria University, Alexandria 21521, Egypt; 2 Department of Clinical Pharmacy, Alexandria University Main Teaching Hospital, Alexandria 21526, Egypt; 3 Human Genetics Department, Medical Research Institute, Alexandria University, Alexandria 21521, Egypt; 4 Department of Infectious Diseases Surveillance, El Amereya Medical Area, Ministry of Health, Alexandria 53330, Egypt; 5 Department of Pharmaceutical and Medicinal Chemistry, Institute of Pharmacy, Freie Universität Berlin, Königin-Luise-Straße 2+4, 14195 Berlin, Germany; 6 Department of Pharmaceutical Chemistry, Faculty of Pharmacy, Alexandria University, Alexandria 21521, Egypt; 7 Department of Medicinal Chemistry, Faculty of Pharmacy, Alamein International University, Alamein 51718, Egypt; 8 Microbiology and Immunology Department, Faculty of Pharmacy and Drug Manufacturing, Pharos University in Alexandria, Alexandria 21648, Egypt

**Keywords:** antibacterial activity, Efavirenz, drug repurposing, in silico binding study

## Abstract

The repurposing potential of Efavirenz (EFV), a clinically established non-nucleoside reverse transcriptase inhibitor, was comprehensively evaluated for its in vitro antibacterial effect either alone or in combination with other antibacterial agents on several Gram-positive clinical strains showing different antibiotic resistance profiles. The binding potential assessed by an in silico study included Penicillin-binding proteins (PBPs) and WalK membrane kinase. Despite the relatively high minimum inhibitory concentration (MIC) limiting the use of EFV as a single antibacterial agent, it exhibits significant synergistic activity at sub-MIC levels when paired with various antibiotics against *Enterococcus* species and *Staphylococcus aureus*. EFV showed restored sensitivity of β-lactams against Methicillin-resistant *S. aureus* (MRSA). It increased the effectiveness of antibiotics tested against Methicillin-sensitive *S. aureus* (MSSA). It also helped to overcome the intrinsic resistance barrier for several antibiotics in *Enterococcus* spp. In silico binding studies aligned remarkably with experimental antimicrobial testing results and highlighted the potential of EFV to direct the engagement of PBPs with moderate to strong binding affinities (pK_a_ 5.2–6.1). The dual-site PBP2 binding mechanism emerged as a novel inhibition strategy, potentially circumventing resistance mutations. Special attention should be paid to WalK binding predictions (pK_a_ = 4.94), referring to the potential of EFV to interfere with essential regulatory pathways controlling cell wall metabolism and virulence factor expression. These findings, in general, suggest the possibility of EFV as a promising lead for the development of new antibacterial agents.

## 1. Introduction

The emergence and dissemination of multi-drug-resistant (MDR) microorganisms are significant public health problems due to the high morbidity and mortality of infections caused by these agents [1]. The advancement of novel antibiotics has significantly declined, resulting in restricted effective treatment alternatives against MDR microorganisms [2]. Most new antibiotics do not have a distinct mechanism of action but are modifications of existing antibiotic classes [3,4].

Drug repositioning or drug repurposing entails discovering novel therapeutic uses for existing medications, leveraging their established safety profiles and pharmacological information to deliver effective treatments for resistant pathogens rapidly [5]. Successful drug repurposing may come with several benefits, including line extension for existing drugs and possible new targets for developing antibiotics [6].

**Scheme 1:**
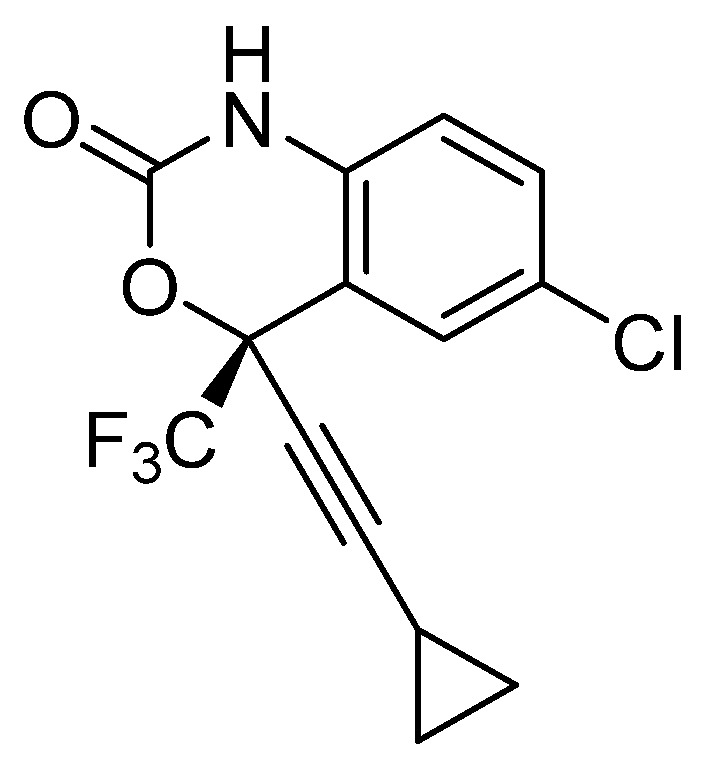
Chemical structure of the non-nucleoside reverse transcriptase inhibitor Efavirenz.

Antiretroviral (ARV) drugs were found to directly impact the disruption of the intestinal microbiota and the selection of MDR microorganisms in treated patients [7,8]. This was attributed to their potential antibacterial activity [9,10]. Among these ARV drugs is Efavirenz (EFV, Scheme 1), which is a non-nucleoside reverse transcriptase inhibitor (NNRTI) that is part of a first-line medication combination in the treatment of human immunodeficiency virus (HIV) infection in adults and adolescents [11]. The documented antibacterial activity renders EFV an appropriate candidate for investigation as a prospective antibacterial agent.

This work aimed to evaluate the in vitro antibacterial effect of EFV alone or combined with other antibacterial drugs on different Gram-positive clinical strains showing different antibiotic resistance profiles. The study also aimed to determine the potential mechanism of action of EFV and its possible effects on bacterial virulence using in silico modeling.

## 2. Results and Discussion

**Figure 1:**
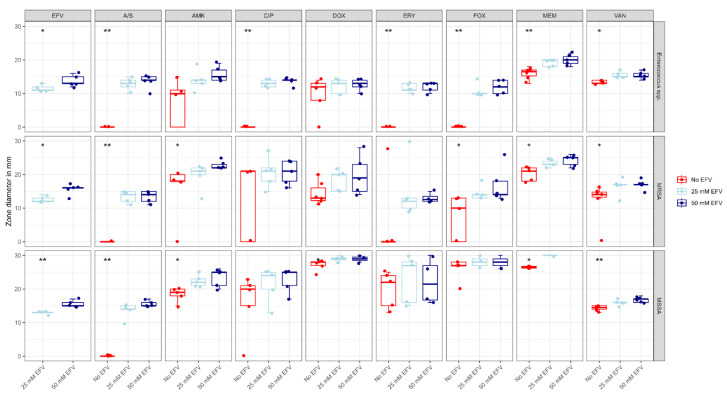
Effect of 25 mM and 50 mM of EFV on the diameter zone for eight antibiotics against *Enterococcus* spp., MRSA, and MSSA isolates. The asterisks indicate statistically significant change, * *p* value < 0.05, ** *p* value < 0.01. Disk diffusion assay showing the antibacterial effect of EFV (25 mM and 50 mM) by the zone of inhibition diameter (mm), alone or in combination with eight selected antibiotics against clinical isolates of *Enterococcus* spp. (*n* = 5), MRSA (*n* = 5), and MSSA (*n* = 5). EFV showed a significant dose-dependent antibacterial effect on the study isolates. EFV also restored sensitivity to antibiotics with no baseline activity. Significant increases in the inhibition zone with one or both concentrations were observed for β-lactams (Meropenem (MEM), Ampicillin/Sulbactam (A/S), Cefoxitine (FOX)) and aminoglycosides (Amikacin (AMK)) in MSSA and/or MRSA, while *Enterococcus* spp. showed a significant change with β-lactams, Vancomycin (VAN), and quinolones (Ciprofloxacin (CIP)) (*p* < 0.05, asterisks). Data represent median ± interquartile range (IQR); statistics: Kruskal–Wallis/Dunn’s test (multi-group) or Wilcoxon rank-sum test (two-group).

In this work, the aim was to assess the antibacterial effect of EFV. Several studies have illustrated the antimicrobial and virulence impact of EFV [12,13]. Upon examination using the disk diffusion method, EFV showed weak antibacterial activity. However, the effect showed a significant increase between the two prepared disk concentrations (25 mM and 50 mM) among all the included Gram-positive bacteria. This suggests a dose-dependent antimicrobial effect of EFV (Figure 1). When investigating the minimum inhibitory concentration (MIC), EFV exhibited an MIC of 16 µg/mL across all tested strains. Nevertheless, the clear wells were sub-cultured and showed no growth, indicating a bactericidal effect of EFV.

Both through disk diffusion and MIC testing, EFV demonstrated clear synergy with the antibiotic in Methicillin-resistant *Staphylococcus aureus* (MRSA) and Methicillin-sensitive *Staphylococcus aureus* (MSSA); however, the synergy is more evident in MSSA. During the disk diffusion assay (Figure 1) MSSA showed a significant increase in inhibition zones for Amikacin (AMK), Vancomycin (VAN), Meropenem (MEM), Ampicillin/Sulbactam (A/S), and Doxycycline (DOX), with an apparent dose-dependent change, especially for MEM. Similarly, MRSA exhibited significant but less pronounced enhancements for AMK, VAN, MEM, and A/S, with smaller increases between 25 and 50 mM of EFV (Table 1).

**Figure 2:**
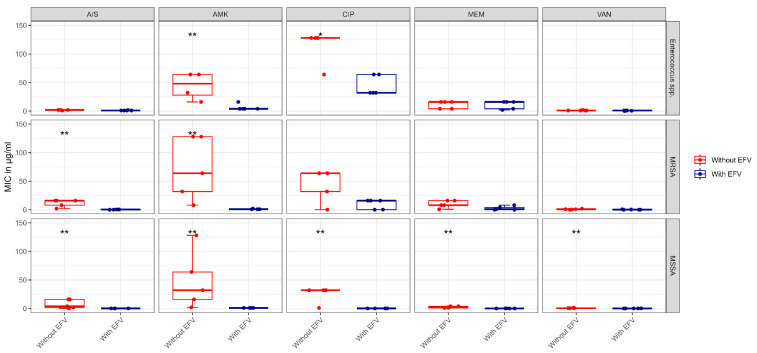
MIC of five antibiotics against *Enterococcus* spp., MRSA, and MSSA isolates without and with ½ × MIC EFV. The asterisks indicate statistically significant change, * *p* value < 0.05, ** *p* value < 0.01. MIC (μg/mL) of five antibiotics against *Enterococcus* spp. (*n* = 5), MRSA (*n* = 5), and MSSA (*n* = 5), tested alone (control) and with ½ × MIC EFV (8 μg/mL). EFV significantly reduced MICs for MSSA (all antibiotics (4–256-fold reduction; *p* < 0.05)), MRSA (AMK (64→1 μg/mL)), and A/S (16→0.5 μg/mL; *p* < 0.05). *Enterococcus*: AMK (64→4 μg/mL; *p* = 0.009) and CIP (128→32 μg/mL; *p* = 0.011). Asterisks denote statistical significance (Wilcoxon rank-sum test). Boxes: median (line), IQR (box), range (whiskers).

The aforementioned findings were further endorsed by the effect of EFV on MIC reduction (Figure 2). All antibiotics showed reduced MICs when augmented with ½ × MIC of EFV, when tested on MRSA and MSSA, with a fold reduction ranging from 4- to 256-fold. However, MRSA demonstrated significant MIC reductions only for AMK and A/S, while MSSA showed a statistically significant decrease for all five antibiotics tested, including VAN, CIP, and MEM (Table 2).

The differences in response may reflect genotypic and phenotypic resistance mechanisms that are unique to MRSA. PBP2a, encoded by mecA, confers high-level resistance to β-lactams by reducing their affinity for PBPs, limiting the synergistic effect of EFV [14]. MSSA, which lacks PBP2a, remains inherently more susceptible to β-lactam antibiotics and can more fully benefit from EFV-mediated enhancement. Despite the intrinsic resistance to β-lactams seen in MRSA, a significant enhancement could be observed in combination with EFV in either the MIC or disk diffusion test. This enhancement in susceptibility was found in combination with A/S and MEM, which surpasses the effect of EFV alone.

Another interesting observation was the difference between the effect of EFV on CIP in *Staphylococci*. MRSA often overexpresses multi-drug efflux pumps, such as NorA, contributing to resistance against fluoroquinolones and other antibiotic classes [15]. However, although an increase in susceptibility to CIP was observed, no statistical significance was given for this combination. This could be explained by EFV only weakly interfering with these transporters; thus, it may not sufficiently enhance intracellular CIP concentrations in MRSA compared to MSSA [16].

In contrast to *Staphylococci*, *Enterococcus* spp. showed more limited and selective synergy with EFV. Disk diffusion testing revealed that EFV restored antibiotic activity in combination such as with CIP, A/S, Erythromycin (ERY), and Cefoxitin (FOX), all of which had 0 mm inhibition zones under control conditions (Figure 1). Significant zone diameter increases were observed for these drugs in combination with EFV, albeit with smaller absolute diameters (e.g., 13–14 mm), and without a clear dose-dependent trend. EFV alone also produced measurable zones (~11–13 mm), indicating intrinsic antibacterial activity against *Enterococci*.

MIC testing confirmed a selective effect of EFV in *Enterococcus* spp., with significant MIC reductions observed only for AMK (64→4 µg/mL, *p* = 0.009) and CIP (128→32 µg/mL, *p* = 0.011). No significant MIC reductions were seen for β-lactams and glycopeptides, in contrast to the consistent enhancements observed in MSSA (Figure 2). *Enterococci* express intrinsic low-level resistance to Penicillin, due to expression of PBP5, which has a low affinity to β-lactams [17,18]. This suggests that the inherent EFV activity in these combinations could explain the observed enhancement of the susceptibility to β-lactams and glycopeptides in *Enterococci*.

On the other hand, the intrinsic resistance of *Enterococci* to aminoglycosides is mainly caused by limited drug uptake. Combining aminoglycosides with cell wall-active agents can lead to disruptions in the cell wall, increasing permeability, and ultimately enabling aminoglycoside influx [19].

The absence of significant synergy with β-lactams and glycopeptides in *Enterococcus* suggests that the drug is more effective in organisms with higher intrinsic susceptibility and has structural barriers to drug entry and action. Nevertheless, the significant restoration of the activity of different antibiotics, especially to CIP and AMK in *Enterococcus* spp. and its restoration in AMK and A/S against both MRSA and MSSA, suggests that EFV may still partially overcome resistance by enhancing permeability. This premise could be explained if EFV is acting on either the cell wall or the cell membrane as a target. This could be further endorsed by the lipophilicity of EFV [20], which can help with insertion into bacterial membranes. Through this premise, WalK, a sensor histidine kinase embedded in the cell membrane, was hypothesized as a potential target [21]. WalK was prioritized as a conserved histidine kinase in the WalKR regulon, governing cell wall metabolism in Gram-positives bacteria. It plays a critical role in sensing cell envelope stress and regulating genes. Also, WalK inhibitors have yielded potent antibacterial activity, making WalK a rational target for synergy studies. Destabilization of this membrane-bound protein may increase permeability, enabling enhanced antibiotic influx [22,23]. This suggested theory may explain how EFV can exert synergistic effects that are common to all tested bacteria, but are more evident in susceptible bacteria like MSSA. The proposed effect on permeability is still more of a hypothesis; however, upcoming studies are needed to confirm this.

The repurposing potential of EFV, a clinically established NNRTI, was comprehensively evaluated against essential bacterial targets using an integrated computational pipeline. The in silico study was conducted only for targets showing promising results in the initial screening. Molecular docking was performed using a Genetic Neural Network-based Interaction Analyzer (GNINA) to assess the binding potential against PBPs1-4 from *S. aureus*, *Enterococcus* spp. (PBPs4-5), and WalK. PBPs were selected as targets given their role in the synthesis of the cell wall and due to their significance as targets of commonly used β-lactam antibiotics. WalK is useful because of its contribution to the metabolism of the cell wall in Gram-positive bacteria. The assessment employed three complementary metrics, Vina affinity (Vina binding Score), CNN affinity (pose reliability), and CNN score (binding pose plausibility), providing a robust evaluation of both binding strength and structural credibility. Cross-validation was performed using the OnionNet2 convolutional neural network (CNN) for binding free energy prediction, enabling quantitative comparison across diverse drug candidates and antibiotic controls (Table 3).

**Figure 3:**
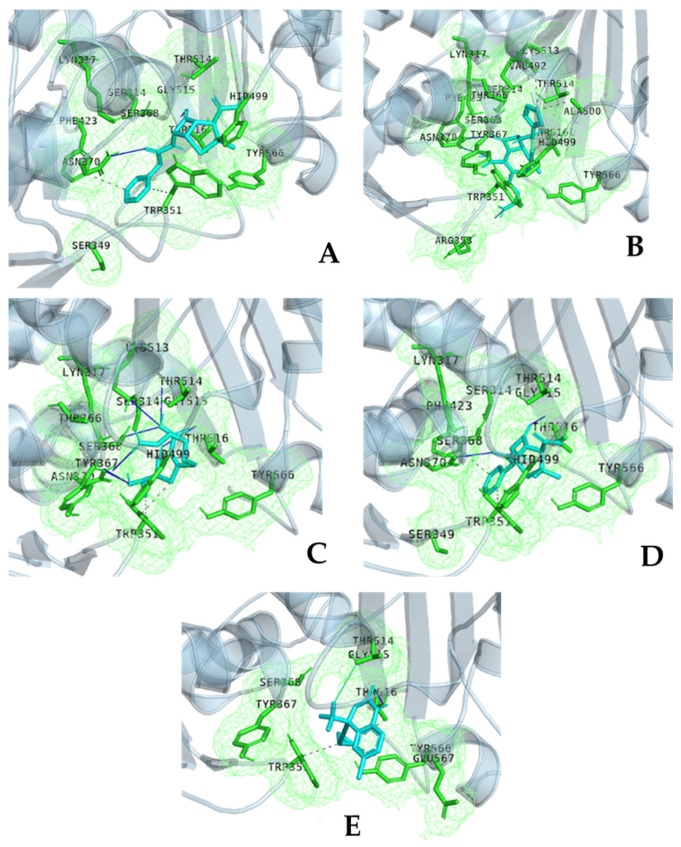
Comparative interaction fingerprints of β-lactam antibiotics and EFV within *S. aureus* PBP1. Panels (**A**–**D**) illustrate the binding modes of different drugs (cyan) (**A**) amoxicillin, (**B**) cefuroxime, (**C**) imipenem, and (**D**) penicillin G within the catalytic pocket of PBP1, characterized by the covalent acylation of the catalytic Ser304 and conserved H-bonding with residues such as Asp267, Gln285, Lys300, and Asn308. These β-lactams also form a salt bridge between their carboxylate moieties and Lys544, stabilizing their transition-state mimicry. Panel (**E**) shows EFV binding in a distal allosteric site, approximately 15 Å from Ser304. EFV is stabilized via hydrophobic interactions with Trp351, Phe423, and Tyr566, H-bonds with Asn370 and Thr516, and a halogen bond between its fluorine atom and Thr514. Green meshes represent interaction fingerprints derived from docking pose analysis, highlighting key pharmacophoric features. While β-lactams uniformly engage the catalytic core, EFV adopts a non-canonical, allosteric binding mode that avoids direct interference with catalytic residues. This mechanistic divergence may underlie the ability of EFV to evade β-lactam resistance mechanisms and support its development as a novel antibacterial scaffold. Cyan represents the ligand (EFV or β-lactams), green meshes denote the pocket perimeter or interaction fingerprints, and grey depicts the protein backbone.

EFV demonstrated substantial binding to PBP1 with a minimum Vina affinity of −7.60 kcal/mol (mean = −6.33 kcal/mol), a CNN affinity minimum of 4.58 kcal/mol, and a CNN score maximum of 0.77. EFV ranked fifth in binding affinity compared to five standard β-lactam antibiotics tested against PBP1. However, it maintained the second-highest CNN score, indicating superior pose confidence relative to its binding energy. Figure 3 simulates the binding modes of the docked ligands within PBP1. Cefuroxime achieved the most potent interaction (−8.74 kcal/mol, CNN score 0.88), followed by Amoxicillin and Penicillin G (both −8.21 kcal/mol, CNN scores 0.90), and Imipenem (−7.77 kcal/mol, CNN score 0.83). The binding affinity gap of 1.14 kcal/mol between EFV and Cefuroxime represents an approximately 6-fold difference in binding constants. Yet, the affinity of EFV remains within the pharmacologically relevant range for enzyme inhibition. Notably, the CNN score of EFV of 0.77 approaches those of established β-lactams, suggesting comparable binding pose reliability despite having a lower thermodynamic affinity. OnionNet2 predicted the binding affinity of EFV to PBP1 as pKa = 5.88, compared to Amoxicillin (pKa = 7.31), Cefuroxime (pKa = 6.98), and Imipenem (pKa = 6.42), confirming a moderate but biologically relevant interaction strength.

**Figure 4:**
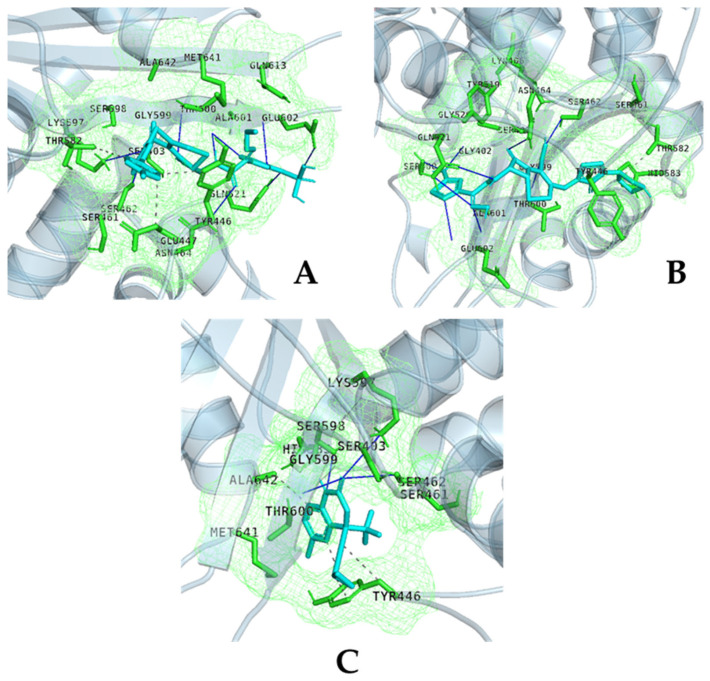
Comparative binding modes of Ceftaroline, Ceftobiprole, and EFV at the catalytic site of *S. aureus* PBP2a. The drug (cyan) (**A**) Ceftaroline engages the catalytic site with 11 H-bonds involving residues such as Thr600, Ser462, Glu602, and Lys597, mimicking the native D-Ala-D-Ala substrate. Hydrophobic interactions with Tyr446 and Thr582 further stabilize the complex, enabling the effective covalent acylation of the catalytic Ser400. (**B**) Ceftobiprole binds similarly but with a streamlined polar network (nine H-bonds), notably forming strong interactions with Ser400, Gln521, and Glu602. It lacks an interaction with Lys597 but compensates through compact hydrophobic engagement with Tyr446. (**C**) EFV adopts a distinct, non-catalytic binding pattern. It forms only four H-bonds (e.g., with Ser462, Ser598) and lacks contacts with Ser400 or Glu602. Instead, it stabilizes through hydrophobic contacts with Tyr446 and Ala642, occupying a peripheral sub-pocket. Green meshes highlight key interaction fingerprints. The divergence in polar and spatial engagement explains the high inhibitory potency of β-lactams versus the limited catalytic interference by EFV. Cyan represents the ligand (EFV or β-lactams), green meshes denote the pocket perimeter or interaction fingerprints, and grey depicts the protein backbone.

**Figure 5:**
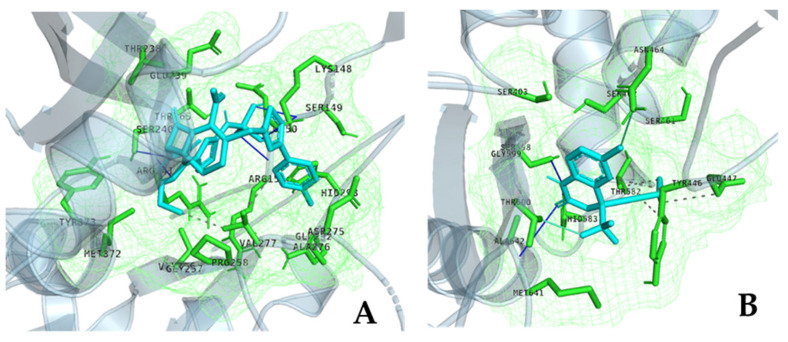
Divergent allosteric binding strategies of Ceftaroline and EFV within *S. aureus* PBP2a. The drug (cyan) (**A**) Ceftaroline anchors to the allosteric site via a polar interaction network, forming six H-bonds with key residues including Lys148, Ser149, Arg151, and Arg241. A bifurcated H-bond at Ser149 and hydrophobic contacts with Val277 stabilize its position near the allosteric–catalytic interface, enabling electrostatic signal transmission toward the active site. (**B**) EFV occupies a deep hydrophobic sub-pocket, engaging in extensive hydrophobic contacts with Tyr446, Glu447, and Thr582. It forms two halogen bonds—between its chlorine atom and Asn464, and between its fluorine atom and Thr600—providing geometric precision and rigid stabilization. Green interaction fingerprints highlight key pharmacophoric regions. The distinct binding topologies underscore mechanistic differences: Ceftaroline relies on polar networks to mediate allosteric regulation. Meanwhile, EFV exploits hydrophobic and halogen bonding to achieve stable non-catalytic site engagement, potentially offering improved resistance evasion and tissue permeability. Cyan represents the ligand (EFV or β-lactams), green meshes denote the pocket perimeter or interaction fingerprints, and grey depicts the protein backbone.

PBP2a catalytic site analysis revealed, for EFV, a minimum Vina affinity of −7.23 kcal/mol (mean = −6.16 kcal/mol), ranking third among the three-ligand test set, including Ceftobiprole and Ceftaroline. Figure 4 illustrates the possible binding modes of the docked ligands with PBP2a. While Ceftobiprole demonstrated the strongest binding (−10.37 kcal/mol) with a remarkable 3.14 kcal/mol advantage over EFV, and Ceftaroline showed robust affinity (−8.92 kcal/mol), EFV maintained a moderate binding with a CNN affinity minimum of 3.75 and a CNN score maximum of 0.54. The OnionNet2 analysis predicted the catalytic site affinity for EFV as pKa = 6.11, compared to Ceftobiprole (pKa = 7.12) and Ceftaroline (pKa = 6.44), positioning EFV within the moderate-to-strong binding range. Remarkably, EFV demonstrated a binding capability to the allosteric site of PBP2a (Figure 5) with a minimum Vina affinity of −6.66 kcal/mol (mean = −6.02 kcal/mol), a CNN affinity minimum of 3.77, and a CNN score maximum of 0.37. Only Ceftaroline among the standard antibiotics showed allosteric site engagement (−8.03 kcal/mol), outperforming EFV by 1.37 kcal/mol. However, the dual-site binding capability of EFV—the simultaneous engagement of both catalytic and allosteric pockets—represents a unique mechanism of action that is absent in most conventional antibiotics. This polypharmacological profile may enable weak inhibition at multiple regulatory points, which is potentially relevant for combination therapies or resistance modulation strategies. The OnionNet2 prediction of EFV for allosteric binding (pKa = 6.11, identical to catalytic site) suggests comparable binding energetics at both sites, supporting the hypothesis of balanced dual-site engagement that could provide sustained enzyme inhibition through multiple interaction points.

**Figure 6:**
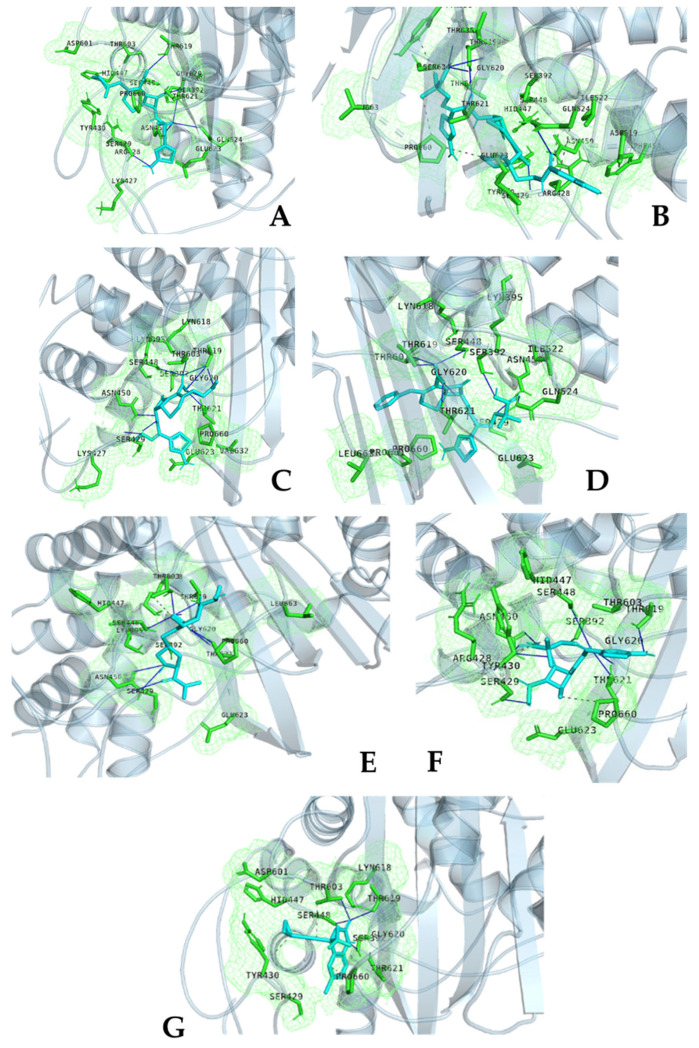
Structural interaction fingerprints of β-lactam and non-β-lactam ligands bound to *S. aureus* PBP3. (**A**–**F**) β-lactam antibiotics (drugs displayed in cyan) demonstrate conserved catalytic engagement via H-bonds with key residues: Ser429, Ser448, Asn450, Thr603, Thr619, and Thr621. These residues mimic the D-Ala-D-Ala binding motif of native peptidoglycan. (**A**) Cefepime forms a strong polar network with backbone residues and engages Glu623. (**B**) Cefiderocol utilizes unique interactions with Tyr636 and Gly620 to stabilize its iron-chelating side chain, while maintaining contacts with Pro660. (**C**) Cefotaxime maintains core H-bonding interactions and engages Thr619/Thr621. (**D**) Ceftazidime exhibits the highest H-bond density (11 bonds), including exclusive interactions with Gln524 and Arg528. (**E**) Meropenem forms a dual H-bond with Asn450 and strong polar contacts with Thr603, features associated with high acylation efficiency. (**F**) Aztreonam interacts with Ser448, Thr621, and Tyr430. (**G**) EFV adopts a distinct, non-catalytic binding pose, stabilized primarily through hydrophobic interactions with Pro660, Tyr430, and Thr603, and forms fewer polar contacts. Green mesh surfaces indicate pharmacophore interaction zones. The conserved catalytic residues represent mutational hotspots (e.g., Thr619, Asn450), while ligands engaging broader or peripheral sites (e.g., Cefiderocol, EFV) may retain efficacy under resistance pressures. These findings suggest ligand-specific strategies to preserve binding in the context of emerging β-lactam resistance. Cyan represents the ligand (EFV or β-lactams), green meshes denote the pocket perimeter or interaction fingerprints, and grey depicts the protein backbone.

PBP3 analysis revealed that for EFV, the binding affinity was −7.53 kcal/mol minimum (mean = −5.42 kcal/mol). EFV maintained biologically relevant binding strength, with superior antibiotics including Cefiderocol (−10.27 kcal/mol), Ceftazidime (−9.25 kcal/mol), Cefepime (−8.79 kcal/mol), Aztreonam (−8.47 kcal/mol), Cefotaxime (−8.45 kcal/mol), and Meropenem (−8.20 kcal/mol), despite ranking last. Figure 6 illustrates the binding interactions of the docked ligands with the active site of PBP3. The 2.74 kcal/mol difference between EFV and Cefiderocol represents a substantial but not prohibitive binding disparity. CNN metrics revealed a score of 0.27 for EFV, which is lower than most β-lactam comparators, yet OnionNet2 predicted moderate binding affinity (pKa = 5.80). Notably, Cefiderocol, despite having the strongest Vina affinity, showed the lowest CNN score (0.13), highlighting the importance of multi-metric evaluation. The intermediate OnionNet2 prediction of EFV places it between Cefiderocol (pKa = 7.48) and Ceftazidime (pKa = 4.83), suggesting moderate inhibitory potential at physiological concentrations.

**Figure 7:**
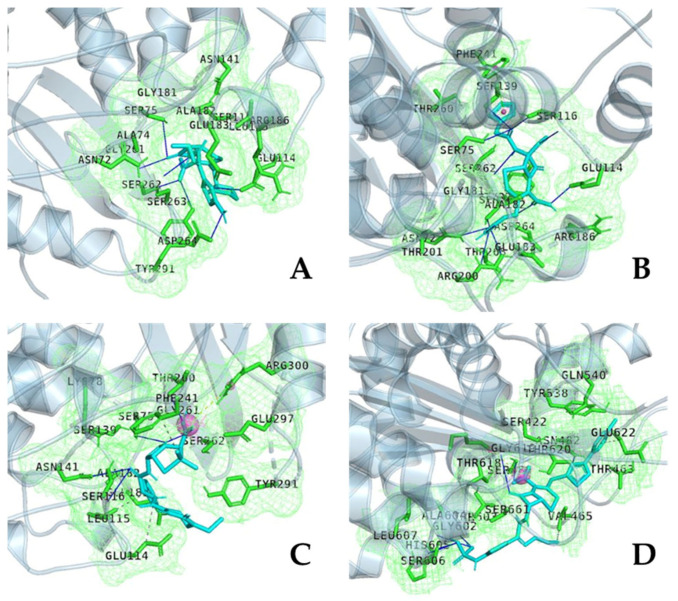
Interaction fingerprints of β-lactam antibiotics and EFA bound to *Pseudomonas aeruginosa* PBP4. (**A**–**C**) β-lactam antibiotics (drugs displayed in cyan) engage the conserved catalytic site centered around Ser75 and Ser262, facilitating acylation through a network of H-bonds. (**A**) Cefepime forms H-bonds with catalytic Ser75, Ser262, and surrounding residues, anchored via Glu114 and stabilized by peripheral contacts including Asn72 and Glu181. (**B**) Cefoxitin exhibits the most extensive interaction network, forming 12 H-bonds, including dual contacts with Arg200 and π-stacking with Phe241. (**C**) Piperacillin combines hydrophobic contacts with Leu115 and Glu114 with polar interactions involving Thr260, Glu297, and Arg300. (**D**) EFV displays a distinct binding mode, stabilized primarily by hydrophobic interactions with Phe241, Leu607, and Val465, and halogen bonding with Tyr291. Its interaction bypasses catalytic residues and centers around a lipophilic sub-pocket, with Arg300 contributing to electrostatic stabilization. Green meshes represent interaction pharmacophores derived from docking pose analysis. Unlike β-lactams that engage in extensive polar interactions for catalytic inhibition, EFV anchors in a peripheral hydrophobic cavity, supporting an allosteric or non-classical inhibition strategy. Cyan represents the ligand (EFV or β-lactams), green meshes denote the pocket perimeter or interaction fingerprints, and grey depicts the protein backbone.

EFV binding to PBP4 yielded a minimum Vina affinity of −7.07 kcal/mol (mean = −5.59 kcal/mol), ranking fourth among the tested set, including Piperacillin, Cefepime, and Cefoxitin. Figure 7 illustrates the possible binding modes of the docked antibiotics compared to EFV within PBP4. EFV recorded a CNN affinity minimum of 3.83 kcal/mol and a CNN score maximum of 0.38. Superior performers included Cefepime (−8.85 kcal/mol, CNN score 0.45), Piperacillin (−8.69 kcal/mol, CNN score 0.56), and Cefoxitin (−8.51 kcal/mol, CNN score 0.56). All standard antibiotics demonstrated 1.44–1.78 kcal/mol binding advantages over EFV, yet maintained comparable CNN score ranges, supporting the structural binding plausibility of EFV. OnionNet2 analysis predicted the affinity of EFV to PBP4 as pKa = 5.31, which is substantially lower than that of Cefepime (pKa = 7.03), Cefoxitin (pKa = 6.79), and Piperacillin (pKa = 7.15). EFV remains within biologically relevant binding ranges despite its lower absolute affinity, making it particularly suitable for combinatorial therapeutic strategies where moderate individual target engagement may provide synergistic enhancement.

**Figure 8:**
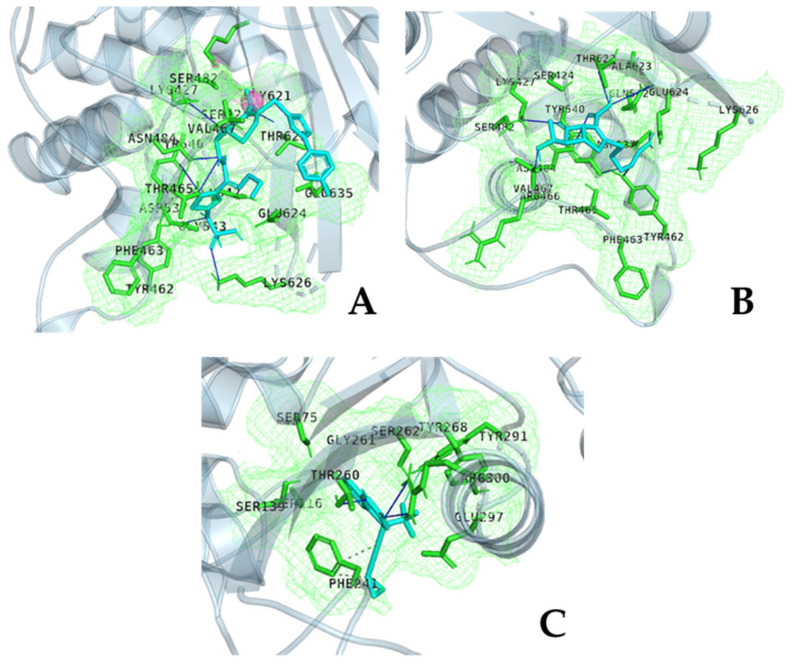
Comparative interaction fingerprints of Ceftaroline, EFV, and Imipenem within the catalytic site of *Enterococcus* PBP4. The drug (cyan) (**A**) Ceftaroline binds the catalytic cleft via H-bonds with Asn484 and Lys427, and uniquely forms a salt bridge with Lys619 to stabilize its carboxylate. Additional polar interactions with Asp537 and Thr622 suggest the dynamic recognition of its β-lactam core. (**B**) Imipenem adopts a similar polar-binding mode, forming H-bonds with catalytic Lys427 and Asn484 and dual interactions involving Tyr462 and Tyr540. Asp537 donates a H-bond to the amine of Imipenem, reflecting its role as a flexible binding mediator. (**C**) EFV retains its canonical halogen bond (fluorine–Tyr291, 3.79 Å) and hydrophobic contacts with Phe241, but diverges from prior PBP4 interactions by engaging Val467 rather than Arg300. Across all ligands, Thr622 emerges as a conserved carboxylate-binding residue. Asp537 acts as a versatile molecular switch, accommodating different scaffolds via the donation and acceptance of H-bonds. Green mesh highlights ligand interaction surfaces derived from docking pose fingerprints, illustrating the distinct chemical strategies used by β-lactams and EFV to modulate PBP4 function. Cyan represents the ligand (EFV or β-lactams), green meshes denote the pocket perimeter or interaction fingerprints, and grey depicts the protein backbone.

EFV demonstrated binding to enterococcal PBP4 with a minimum Vina affinity of −6.79 kcal/mol (mean = −5.37 kcal/mol), ranking third among the three tested ligands. Figure 8 demonstrates the possible binding interactions of EFV compared to the docked antibiotics within PBP4. Ceftaroline exhibited superior binding (−8.89 kcal/mol) with a 2.10 kcal/mol advantage, while Imipenem showed moderate superiority (−7.07 kcal/mol). CNN metrics revealed a minimum affinity of 3.24 kcal/mol and a maximum score of 0.27 for EFV, indicating moderate pose confidence. OnionNet2 predicted binding affinity as pKa = 5.20, compared to Ceftaroline (pKa = 6.95) and Imipenem (pKa = 6.66). The weaker binding to enterococcal PBP4 correlates with experimental observations of limited β-lactam synergy in *Enterococcus* ssp., supporting computational predictions of reduced EFV efficacy against enterococcal cell wall synthesis machinery.

**Figure 9:**
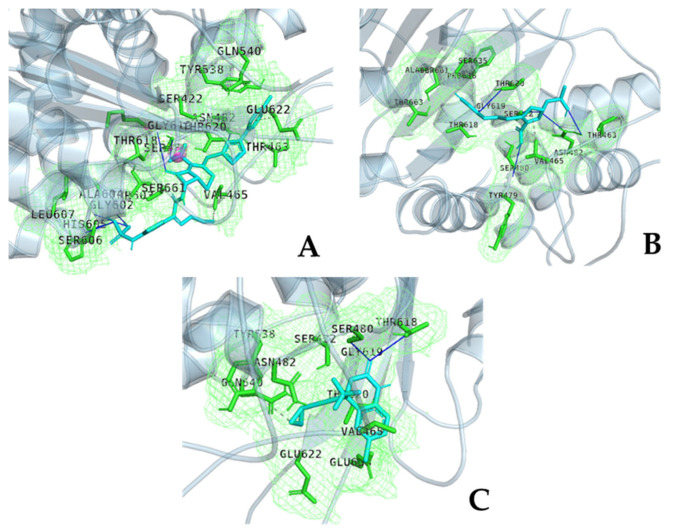
Differential binding profiles of Ceftaroline, Imipenem, and EFV to the catalytic pocket of *Enterococcus* PBP5. The drug (cyan) (**A**) Ceftaroline forms a dense interaction network centered on catalytic Ser480 (H-bond distance: 2.58 Å) and engages Lys617 via a salt bridge that anchors its carboxylate. Additional stabilization is provided through interactions with Asn482, Thr465, Glu622, and a conserved hydrophobic pocket around Val465. (**B**) Imipenem also targets Ser480 (H-bond distance: 3.41 Å) and engages Asn482 and Gly619 through polar interactions. It uniquely contacts Tyr479 and Phe636 through π-stacking interactions, compensating for the absence of a salt bridge with Lys617. (**C**) EFV displays a minimalist binding mode, forming two H-bonds—one with Ser480 and another with Thr618—while relying on hydrophobic contacts with Val465 and reduced engagement with canonical catalytic residues. Green mesh regions represent ligand–receptor interaction surfaces derived from docking pose analysis. The conserved Val465 hydrophobic sub-pocket serves as a structural anchor across all ligands. At the same time, selectivity toward catalytic residues and salt bridge interactions distinguishes β-lactam inhibitors from non-classical scaffolds such as EFV. Cyan represents the ligand (EFV or β-lactams), green meshes denote the pocket perimeter or interaction fingerprints, and grey depicts the protein backbone.

EFV binding to PBP5 yielded a −6.86 kcal/mol minimum affinity (mean = −5.70 kcal/mol), achieving the second rank among the three ligands tested. Figure 9 shows comparative binding modes of EFV, Imipenem, and Ceftaroline. EFV outperformed Imipenem (−6.73 kcal/mol) by a narrow 0.13 kcal/mol margin, while Ceftaroline maintained superiority (−8.39 kcal/mol). A CNN affinity minimum of 3.90 kcal/mol and a score of 0.47 indicated moderate-to-good pose reliability, supported by an OnionNet2 prediction of pKa = 5.47. This represents the strongest relative performance of EFV among enterococcal targets, achieving competitive binding that is comparable to established carbapenems. The superior performance at PBP5 versus PBP4 may reflect structural differences in the architecture of the binding pocket, potentially explaining the experimental selective antimicrobial effects.

**Figure 10:**
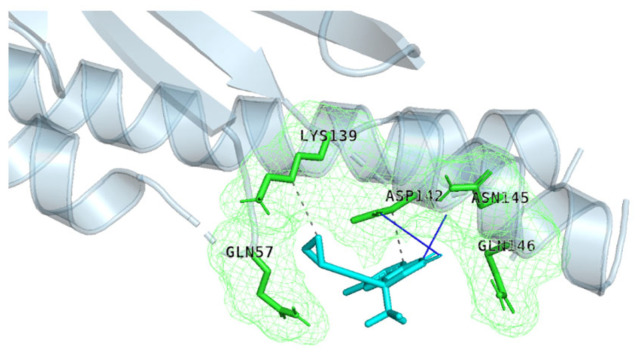
Binding fingerprint of EFV at the sensor domain of *S. aureus* WalK histidine kinase. EFV (drug displayed in cyan) engages a defined pocket near the dimerization interface of the kinase, anchored by a dual H-bonding network with Asp142. Specifically, Asp142 donates a H-bond to the O2 group of the ligand (H–A distance: 3.58 Å) and accepts a bond from the amino group of the ligand (D–A distance: 3.07 Å). Surrounding hydrophobic contacts with Lys139 and Asp142 aliphatic carbons stabilize the interaction, forming a compact binding environment. Additional polar residues—Gln57, Asn145, and Gln146—form a perimeter around the binding cleft. The interaction fingerprint (green mesh) highlights key ligand–receptor contacts, suggesting that EFV may allosterically interfere with WalK autophosphorylation or signaling transmission. This binding mode reveals the potential for repurposing EFV as a histidine kinase inhibitor targeting bacterial two-component systems. Cyan represents the ligand (EFV or β-lactams), green meshes denote the pocket perimeter or interaction fingerprints, and grey depicts the protein backbone.

EFV demonstrated binding to the WalK regulatory protein with a minimum Vina affinity of −5.52 kcal/mol (mean = −4.29 kcal/mol), a CNN affinity minimum of 4.00 kcal/mol, and a notably high CNN score maximum of 0.75. Figure 10 illustrates the binding fingerprint of EFV at the sensor domain of *S. aureus* WalK histidine kinase. OnionNet2 predictions confirmed this similarity: EFV (pKa = 5.27) and Floxuridine (pKa = 5.24) showed nearly identical binding free energies. This convergent binding pattern suggested WalK as a viable target for drug repositioning strategies, potentially offering alternative inhibition mechanisms beyond traditional cell wall synthesis disruption. The high CNN scores (0.75) indicated reliable binding pose predictions, strengthening confidence in the potential of targeting WalK. This regulatory protein pathway may provide opportunities for combination therapy enhancement through simultaneous cell wall synthesis and regulatory network disruption.

The OnionNet2 analysis confirmed a broad-spectrum binding capability of EFV, with the strongest predicted affinities for *S. aureus* targets including PBP2 (both catalytic and allosteric sites), PBP1, moderate–strong binding to PBP3, moderate binding to PBP4, and the regulatory targeting of WalK kinase. For *Enterococcus* ssp., EFV demonstrated moderate-to-strong binding to PBP5 and moderate binding to PBP4. This binding hierarchy correlates with experimental synergy patterns, with the most potent effects observed in *S. aureus* (comprehensive PBP1-4 and WalK engagement) and selective moderate effects in *Enterococci* (PBP4-5 engagement).

Cross-method validation between GNINA Vina scores and OnionNet2 pKa predictions was assessed using Pearson correlation analysis. The correlation coefficient (r) was calculated using the following equation:

(1) $$r=\Sigma [(xi-\bar{x})(yi-\bar{y})]/\surd [\Sigma {\left(xi-\bar{x}\right)}^{2}\Sigma {\left(yi-\bar{y}\right)}^{2}]$$

Xi and yi represent paired binding affinity values from GNINA and OnionNet2 methods, respectively, and $\bar{x}$ and $\bar{y}$ are their corresponding means. The coefficient of determination was calculated as R^2^ = r^2^. The Python 3.10 validation script employed three independent calculation methods (manual implementation, SciPy, and NumPy) to ensure computational accuracy and included comprehensive statistical analysis with visualization.

Analysis of EFV binding data across eight protein targets revealed a moderate correlation (r = 0.555, R^2^ = 0.308), indicating that the methods capture complementary aspects of protein–ligand binding. While perfect correlation was not observed, both computational approaches consistently identified EFV as exhibiting a moderate binding affinity across multiple essential targets, supporting the conclusion of genuine multi-target engagement potential rather than a computational artifact. The correlation analysis was validated through triple-method verification, confirming mathematical accuracy and statistical interpretation.

While GNINA and OnionNet2 provide robust binding predictions, this study is limited because it was conducted on static binding models, so molecular dynamics simulations are needed in a future approach for conformational flexibility assessment. Also, the lipophilic effects of EFV on the cell membrane require specialized modeling approaches [24,25].

The computational binding predictions align remarkably with experimental antimicrobial testing results. EFV demonstrated a uniform MIC of 16 µg/mL across all tested Gram-positive isolates (*S. aureus*, MRSA/MSSA, and *Enterococcus* spp.), indicating consistent moderate antimicrobial activity independent of species-specific resistance mechanisms. Sub-culture analysis confirmed bactericidal activity, supporting computational predictions of functionally relevant binding interactions. However, the approved clinical dosing of EFV only achieves ~1–4 µg/mL in plasma [26], far below its MIC. Thus, the true potential of EFV is given by acting at sub-inhibitory levels to potentiate other antibiotics rather than as a primary agent.

The pronounced synergistic effects observed in *S. aureus* isolates directly correlate with computational multi-target binding predictions. MSSA demonstrated MIC reductions when EFV was combined with standard antibiotics. For instance, MRSA showed selective but significant synergy for AMK and A/S, consistent with computational predictions suggesting that the moderate binding of EFV may overcome specific resistance mechanisms (PBP2a-mediated β-lactam resistance). Moreover, computational analysis reveals the capability of EFV for direct PBP engagement across multiple essential targets, with binding affinities ranging from moderate to strong (pKa 5.2–6.1). The dual-site PBP2 binding mechanism represents a novel inhibition strategy, potentially circumventing single-site resistance mutations. While individual target binding remains weaker than optimized β-lactams, simultaneous multi-target engagement may provide cumulative inhibitory effects exceeding single-target potency. Also, WalK binding predictions (pKa = 4.94) suggest that EFV may interfere with essential regulatory pathways controlling cell wall metabolism and virulence factor expression. This regulatory disruption could sensitize bacteria to conventional antibiotics through multiple downstream effects, explaining broad-spectrum synergistic enhancement beyond direct PBP inhibition.

Drug repurposing is an effective method for accelerating the development of antimicrobials. EFV is a well-known NNRTI used in HIV therapy and has drawn attention for its antibacterial properties. Strong safety and pharmacokinetic data from its extensive clinical use enable a faster regulatory trajectory and an ideal dosage plan. Computational analysis has shown the ability of EFV to target various bacterial PBPs while interacting with membrane structures. This hypothesized dual mechanism can prohibit bacteria from gaining resistance, giving a useful delay in the evolution of resistance. However, compared to its acceptable plasma C_max_ (4 µg/mL), its high minimum inhibitory concentration (MIC = 16 µg/mL) limits its use as a monotherapy drug [26].

EFV exhibits significant activity at sub-MIC levels when combined with other antimicrobial agents. In combination with A/S and AMK, EFV showed a restored sensitivity of β-lactams against MRSA. Similarly, it increased the effectiveness of almost every antibiotic tested against MSSA. In addition, it helped AMK and CIP overcome the intrinsic resistance barrier in *Enterococcus* spp. These findings indicate that EFV offers promising use as an adjunctive agent in targeted combination therapy against resistant Gram-positive bacteria. While limited in solo antimicrobial use, it still provides an interesting scaffold for future antimicrobial development and new target discovery.

## 3. Materials and Methods

### 3.1. Bacterial Isolates and Culture Conditions

There was a total of 15 bacterial isolates of two different bacterial species, five *Enterococci* spp. and ten *S. aureus*, among which five isolates were identified as Methicillin-resistant *S. aureus* (MRSA). The isolates were collected from clinical samples received from the microbiology lab of the Medical Research Institute, Alexandria University, Egypt. All isolates were sub-cultured on blood agar and incubated at 37 °C for 18–24 h to ensure viability and purity. The isolates and their antibiotic susceptibility were initially assessed using the VITEK 2 compact system (bioMérieux, Craponne, France). The isolates were stored in 20% glycerol at −20 °C for further use.

### 3.2. Susceptibility Testing Using the Disk Diffusion and Broth Microdilution Method

The effect of EFV on the susceptibility of the isolates to selected antibiotics (Amikacin (AMK), Vancomycin (VAN), Ciprofloxacin (CIP), Meropenem (MEM), Ampicillin/Sulbactam (A/S), Doxycycline (DOX), Cefoxitin (FOX), and Erythromycin (ERY)) was assessed using the standard disk diffusion (Kirby–Bauer) method. A standardized bacterial suspension (equivalent to 0.5 McFarland) was prepared in sterile nutrient broth. A volume of 100 µL of the bacterial suspension was uniformly spread onto Mueller–Hinton plates. A sterile empty disk with a 6 mm diameter was used to load 10 µL of EFV solution of different concentrations (25 mM and 50 mM) to assess the antimicrobial effect of EFV. The inhibition zones of antibiotic disks were measured alone or in combination with EFV by loading 10 µL of the prepared EFV concentrations on the antibiotic disks. The plates were incubated at 37 °C for 18 h. The zones of inhibition were measured in millimeters in three different directions. The average was calculated and interpreted according to Clinical and Laboratory Standards Institute Guidelines [27,28].

The minimum inhibitory concentration (MIC) of EFV and the selected antibiotics, alone and in combination, were determined using a broth microdilution in 96-well microtiter plates. Two-fold serial dilutions of each antibiotic were prepared in sterile Mueller–Hinton Broth. Each well contained a final volume of 100 µL containing 50 µL of the prepared antibiotic concentrations and 50 µL (10^5^ CFU/mL) of the isolate suspension. The following antibiotics were selected for the MIC assay: AMK, VAN, CIP, MEM, and A/S. The tested antibiotics were repeated twice for each isolate alone and combined with half the MIC of EFV (8 µg/mL). Both positive (media + dimethyl sulfoxide (DMSO) at the highest concentration used + bacteria) and negative (media + DMSO) controls were included in each raw. The plates were incubated at 37 °C for 18 h, and the MIC was recorded as the lowest antibiotic concentration that showed no visible bacterial growth [27]. Each clear well was cultured on Mueller–Hinton agar plates to determine the bactericidal effect.

### 3.3. Formatting of Mathematical Components

Molecular docking simulations were performed using GNINA (v1.3), a fork of smina, and AutoDock Vina. GNINA workflow utilizes convolutional neural networks, where ligand sampling is carried out via a chain of Markov chain Monte Carlo (MCMC) chains that perturb the ligand in the specified binding site. After sampling, poses are scored and ranked [29,30,31]. The protein structure was retrieved from the Protein Data Bank (PDB). Then structures were cleaned using Open Babel (v3.1.1) by removing waters, solvent molecules, residuals, or unwanted ligands, and adding polar hydrogens [29,30,31]. Protein targets were prepared for GNINA using the Meeko: interface of AutoDock (accessed on 30 April 2025: https://github.com/forlilab/Meeko) employing meeko_receptor_prepare.py. Afterwards, ligands were obtained as SDF files, converted into 3D coordinates via Open Babel, and prepared using meeko_ligand_prepare.py.

Docking grids were centered on the coordinates of co-crystallized ligands, as identified in each PDB structure. To ensure comprehensive and accurate coverage of the binding site, the Protein–Ligand Interaction Profiler (PLIP) was utilized to characterize key interaction residues and pocket coordinates, which were then used to refine GNINA grid placement [32]. GNINA docking runs were performed with an exhaustiveness parameter of 16 and configured to output the top 20 binding poses per ligand. Ligand conformations were sampled via MCMC perturbations within the binding pocket, as implemented in GNINA. Docked poses were ranked using the CNN scoring function of GNINA, which evaluates binding affinity based on atomic grid features and spatial arrangement. The top-ranked poses for each ligand were selected for further analysis and visualization. Finally, docked poses output from GNINA were processed using Meeko and an in-house Bash script. This procedure separated each docking pose into individual PDB files, parsing associated binding affinities, CNN scoring, and CNN-predicted affinities.

Binding affinity predictions for the GNINA-docked protein–ligand complexes were performed using OnionNet2, a convolutional neural network designed to estimate binding affinities by capturing spatially resolved protein–ligand interactions [33]. OnionNet2 encodes interaction features within concentric radial layers, or “onion shells”, around each ligand atom, providing a robust framework for quantifying binding events based on atomic contact distributions. The feature vectors were generated using the feature.py module, applying the default onion shell radius and atom-type definitions specified in the original OnionNet2 model architecture. Binding affinity inference was conducted using the pre-trained OnionNet2 model via the predict.py script, yielding predicted affinity scores in pK units for each ligand pose. This pipeline allowed for a comparative assessment between CNN-derived scoring functions of GNINA and OnionNet2-predicted binding affinities.

Two complementary approaches were employed to characterize and visualize protein–ligand interactions in the docked complexes. First, the PLIP was utilized to map non-covalent interactions within each docking pose. Docked complexes were parsed pose-by-pose using a custom Bash script to split GNINA output files into separate receptor–ligand PDB files. These files were then input into PLIP, which automatically detected key interactions, including H-bonds, hydrophobic contacts, π–π stacking, salt bridges, and metal coordination, based on geometric and distance criteria. PLIP output provided detailed interaction profiles for each pose, facilitating residue-level mapping and comparative analysis between ligand binding orientations.

## 4. Conclusions and Future Directions

EFV exhibits significant synergistic activity at sub-MIC levels when paired with various antibiotics against *Enterococcus* spp. and *S. aureus*. In silico binding studies align remarkably with experimental antimicrobial testing results and highlight the potential of EFV to direct PBPs engagement with moderate to strong binding affinities (pKa 5.2–6.1). The dual-site PBP2 binding mechanism emerges as a novel inhibition strategy, potentially circumventing resistance mutations. Although the single target binding profile of EFV remains weaker than that of β-lactams, its simultaneous multitarget engagement capacity may provide cumulative inhibitory effects. Special attention should be paid to WalK binding predictions (pKa = 4.94), referring to the potential of EFV to interfere with essential regulatory pathways controlling cell wall metabolism and virulence factor expression. EFV has limited efficacy as a standalone antibiotic due to its high MIC (16 µg/mL) compared to its low achievable plasma levels (1–4 µg/mL). However, it shows strong potential as an adjunct, enhancing the activity of other antibiotics at sub-inhibitory concentrations. These findings suggest the potential of EFV as a promising lead for developing new antibacterial agents. Future studies should be directed to conducting an in vitro study to confirm the effect of EFV on bacterial permeability. Also, an in vivo study is required to evaluate the impact of EFV in re-sensitizing resistant Gram-positive strains to different clinically used antibiotics, besides optimizing the EFV dose for the repurposed use. Studies should be extended to address the evaluation of the anti-virulence effect of EFV against Gram-positive bacteria. Detailed characterization of bactericidal kinetics and comprehensive experimental validation for target prediction are required to better understand the EFV mechanism of action.

## Figures and Tables

**Table 1:** Effect of 25 mM and 50 mM of EFV alone and in combination with eight clinically common antibiotics on the diameter zone against *Enterococci* spp., MRSA, and MSSA.

Organism	Antibiotic	Zone Diameter	25 mM Efavirenz	50 mM Efavirenz	Control	*p*
*Enterococci* spp.	Amikacin	Median (IQR)	14.0 (13.0 to 14.0)	15.0 (14.0 to 17.0)	10.0 (0.0 to 11.0)	0.061
	Vancomycin	Median (IQR)	15.0 (15.0 to 16.0) ^a^	15.0 (15.0 to 16.0) ^b^	13.0 (13.0 to 14.0) ^a,b^	0.01 *
	Ciprofloxacin	Median (IQR)	13.0 (12.0 to 14.0) ^a^	14.0 (14.0 to 14.0) ^b^	0.0 (0.0 to 0.0) ^a,b^	0.004 *
	Meropenem	Median (IQR)	20.0 (18.0 to 20.0) ^a^	20.0 (19.0 to 21.0) ^b^	17.0 (15.2 to 17.0) ^a,b^	0.006 *
	Ampicillin/Sulbactam	Median (IQR)	13.0 (12.0 to 14.0) ^a^	14.0 (14.0 to 15.0) ^b^	0.0 (0.0 to 0.0) ^a,b^	0.006 *
	Doxycycline	Median (IQR)	13.0 (10.0 to 14.0)	13.0 (12.0 to 14.0)	12.0 (8.0 to 13.0)	0.555
	Cefoxitin	Median (IQR)	10.0 (10.0 to 10.0) ^a^	12.0 (10.0 to 14.0) ^b^	0.0 (0.0 to 0.0) ^a,b^	0.004 *
	Erythromycin	Median (IQR)	11.0 (11.0 to 13.0) ^a^	13.0 (11.0 to 13.0) ^b^	0.0 (0.0 to 0.0) ^a,b^	0.006 *
	EFV	Median (IQR)	11.0 (11.0 to 12.0)	13.0 (13.0 to 15.0)	-	0.032 *
MRSA	Amikacin	Median (IQR)	21.0 (20.0 to 22.0)	22.0 (22.0 to 23.0) ^a^	18.0 (18.0 to 20.0) ^a^	0.01 *
	Vancomycin	Median (IQR)	17.0 (17.0 to 17.0)	17.0 (17.0 to 17.0) ^a^	14.0 (13.2 to 15.0) ^a^	0.031 *
	Ciprofloxacin	Median (IQR)	21.0 (18.0 to 22.0)	21.0 (18.0 to 24.0)	21.0 (0.0 to 21.0)	0.456
	Meropenem	Median (IQR)	23.0 (23.0 to 24.0)	25.0 (23.0 to 25.0) ^a^	21.0 (18.0 to 22.0) ^a^	0.014 *
	Ampicillin/Sulbactam	Median (IQR)	14.0 (12.0 to 15.0) ^a^	14.0 (12.0 to 15.0) ^b^	0.0 (0.0 to 0.0) ^a,b^	0.007 *
	Doxycycline	Median (IQR)	20.0 (15.0 to 20.0)	19.0 (15.0 to 23.0)	13.0 (13.0 to 17.0)	0.092
	Cefoxitin	Median (IQR)	14.0 (13.0 to 14.0)	14.0 (14.0 to 18.0) ^a^	10.0 (0.0 to 13.0) ^a^	0.017 *
	Erythromycin	Median (IQR)	12.0 (10.0 to 13.0)	13.0 (12.0 to 15.0)	0.0 (0.0 to 0.0)	0.083
	EFV	Median (IQR)	12.0 (12.0 to 13.0)	16.0 (16.0 to 16.0)	-	0.018 *
MSSA	Amikacin	Median (IQR)	22.0 (21.0 to 23.0) ^a^	25.0 (21.0 to 25.0) ^b^	19.0 (18.0 to 20.0) ^a,b^	0.012 *
	Vancomycin	Median (IQR)	16.0 (16.0 to 16.0)	17.0 (16.0 to 17.0) ^a^	15.0 (14.0 to 15.0) ^a^	0.004 *
	Ciprofloxacin	Median (IQR)	24.0 (20.0 to 25.0)	25.0 (21.0 to 25.0)	20.0 (15.0 to 21.0)	0.191
	Meropenem	Median (IQR)	35.0 (35.0 to 36.0)	41.0 (36.0 to 42.0) ^a^	33.0 (27.0 to 33.0) ^a^	0.036 *
	Ampicillin/Sulbactam	Median (IQR)	14.0 (14.0 to 15.0) ^a^	15.0 (15.0 to 16.0) ^b^	0.0 (0.0 to 0.0) ^a,b^	0.002 *
	Doxycycline	Median (IQR)	29.0 (29.0 to 30.0)	30.0 (29.0 to 35.0) ^a^	28.0 (27.0 to 28.0) ^a^	0.02 *
	Cefoxitin	Median (IQR)	30.0 (28.0 to 31.0)	31.0 (30.0 to 33.0)	27.0 (27.0 to 28.0)	0.109
	Erythromycin	Median (IQR)	27.0 (16.0 to 28.0)	26.0 (17.0 to 30.0)	22.0 (15.0 to 24.0)	0.285
	Efavirenz	Median (IQR)	13.0 (13.0 to 13.0)	15.0 (15.0 to 16.0)	-	0.006 *

For ≥3 group comparisons, the Kruskal–Wallis test was followed by the Dunn’s test for pairwise comparisons between groups if the result was significant. For two-group comparisons, the Wilcoxon rank-sum test was used. * Refers to statistically significant differences between groups. Common superscript letters indicate statistically significant differences between groups.

**Table 2:** Differences in MIC in μg/mL for five antibiotics against *Enterococcus* spp., MRSA, and MSSA isolates after treating without and with EFV.

Organism	Antibiotic	MIC in μg/mL	With Efavirenz **	Control	*p*
*Enterococcus* spp.	Amikacin	Median (IQR)	4.0 (4.0 to 4.0)	64.0 (32.0 to 64.0)	0.009 *
	Vancomycin	Median (IQR)	1.0 (0.5 to 1.0)	1.0 (1.0 to 1.0)	0.095
	Ciprofloxacin	Median (IQR)	32.0 (32.0 to 64.0)	128.0 (128.0 to 128.0)	0.011 *
	Meropenem	Median (IQR)	16.0 (4.0 to 16.0)	16.0 (4.0 to 16.0)	0.811
	Ampicillin/sulbactam	Median (IQR)	1.0 (1.0 to 1.0)	2.0 (2.0 to 2.0)	0.072
MRSA	Amikacin	Median (IQR)	1.0 (1.0 to 1.0)	64.0 (32.0 to 128.0)	0.007 *
	Vancomycin	Median (IQR)	0.25 (0.062 to 0.5)	1.0 (0.5 to 1.0)	0.136
	Ciprofloxacin	Median (IQR)	16.0 (0.25 to 16.0)	64.0 (32.0 to 64.0)	0.053
	Meropenem	Median (IQR)	1.0 (0.062 to 4.0)	8.0 (8.0 to 16.0)	0.055
	Ampicillin/sulbactam	Median (IQR)	0.5 (0.125 to 0.5)	16.0 (8.0 to 16.0)	0.007 *
MSSA	Amikacin	Median (IQR)	1.0 (1.0 to 1.0)	32.0 (16.0 to 64.0)	0.005 *
	Vancomycin	Median (IQR)	0.062 (0.062 to 0.125)	0.5 (0.5 to 1.0)	0.007 *
	Ciprofloxacin	Median (IQR)	0.125 (0.125 to 0.125)	32.0 (32.0 to 32.0)	0.005 *
	Meropenem	Median (IQR)	0.062 (0.062 to 0.062)	2.0 (1.0 to 4.0)	0.007 *
	Ampicillin/sulbactam	Median (IQR)	0.125 (0.125 to 0.125)	4.0 (2.0 to 16.0)	0.005 *

IQR: interquartile range. Wilcoxon rank-sum test. * Refers to statistically significant differences between groups. ** MIC of EFV as a single agent was 16 μg/mL among all tested isolates.

**Table 3:** Predicted binding affinities (Vina, CNN) in kcal/mol along with CNN scores and OnionNet2-predicted pKa values for various β-lactam antibiotics and EFV complexed with PBPs1–5 and WalK kinase in *S. aureus* and *Enterococcus* spp.

Strain	Protein	Ligand	Vina Affinity (kcal/mol)	CNN-Score	CNN Affinity (kcal/mol)	OnionNet2 Predicted pKa
*S. aureus*/MRSA and MSSA	PBP1	Amoxacillin	−8.21	0.3784	5.141	8.769
	PBP1	Cefuroxime	−8.74	0.3964	5.074	7.090
	PBP1	Imipenem	−7.77	0.6219	4.950	7.066
	PBP1	penciling	−8.21	0.3784	5.141	8.769
	PBP1	Efavarinz	−7.60	0.5798	5.441	6.078
	PBP2_Allostric Site	Ceftaroline	−8.03	0.2138	6.459	6.435
	PBP2_Allostric Site	Efavarinz	−6.66	0.2110	4.377	5.877
	PBP2_Catalytic Site	Ceftaroline	−8.92	0.2885	6.501	5.903
	PBP2_Catalytic Site	Ceftobiprole	−10.37	0.1105	5.563	6.478
	PBP2_Catalytic Site	Efavarinz	−7.23	0.1569	4.229	5.932
	PBP3	Aztronem	−8.47	0.1565	4.867	5.254
	PBP3	Cefepime	−8.79	0.1567	4.929	6.157
	PBP3	Cefiderocol	−10.27	0.0798	5.258	7.480
	PBP3	Cefotaxime	−8.45	0.3284	4.858	4.903
	PBP3	Ceftazidime	−9.25	0.4112	5.909	6.523
	PBP3	Meropnem	−8.20	0.4011	4.474	8.546
	PBP3	Efavarinz	−7.53	0.1576	4.385	5.741
	PBP4	Cefepime	−8.85	0.4317	5.467	4.829
	PBP4	Cefoxtitin	−8.51	0.2775	5.290	7.523
	PBP4	Piperacillin	−8.69	0.2913	5.740	6.796
	PBP4	Efavarinz	−7.07	0.2088	4.654	6.775
	WalK	Efavarinz	−5.52	0.7506	5.217	5.349
*Enterococcus* spp.	PBP4	Ceftaroline	−8.89	0.0503	5.651	6.771
	PBP4	Imipenem	−7.07	0.1836	3.829	5.082
	PBP4	Efavarinz	−6.79	0.0874	3.243	6.775
	PBP5	Ceftaroline	−8.39	0.0463	5.214	6.489
	PBP5	Imipenem	−6.73	0.1726	3.427	7.654
	PBP5	Efavarinz	−6.86	0.2015	4.208	6.568

## Data Availability

The dataset is available upon request from the corresponding author A.N.A.

## Abbreviations

AMK	Amikacin
ARV	Antiretroviral
A/S	Ampicillin/Sulbactam
CIP	Ciprofloxacin
CNN	Convolutional Neural Network
DMSO	Dimethyl Sulfoxide
DOX	Doxycycline
EFV	Efavirenz
ERY	Erythromycin
FOX	Cefoxitin
GNINA	Genetic Neural Network-Based Interaction Analyzer
HIV	Human Immunodeficiency Virus
IQR	Interquartile Range
MCMC	Markov Chain Monte Carlo
MDR	Multi-Drug Resistant
MIC	Minimum Inhibitory Concentration
MEM	Meropenem
MRSA	Methicillin-Resistant *Staphylococcus aureus*
MSSA	Methicillin-Sensitive *Staphylococcus aureus*
NNRTI	Non-Nucleoside Reverse Transcriptase Inhibitor
PBP	Penicillin-Binding Protein
PDB	Protein Data Bank
PLIP	Protein–Ligand Interaction Profiler
VAN	Vancomycin
